# A homolog of methionine γ-lyase is required for biofilm development in the cyanobacterium *Synechococcus elongatus*

**DOI:** 10.1007/s11274-025-04712-0

**Published:** 2025-11-25

**Authors:** Eli Zecharia, Linor Shalev, Eleonora Sendersky, Jennifer I C Benichou, Susan S Golden, Rakefet Schwarz

**Affiliations:** 1 The Mina and Everard Goodman Faculty of Life Sciences, Bar-Ilan University, Ramat-Gan, 5290002 Israel; 2 Division of Biological Sciences, University of California, San Diego, La Jolla, CA 92093 USA; 3 Center for Circadian Biology, University of California, San Diego, La Jolla, CA 92093 USA

**Keywords:** Biofilm, Biofilm-matrix, Cyanobacteria, Methionine γ-lyase, *Synechococcus elongatus* sp. PCC 7942, Type IV pilus assembly complex

## Abstract

Bacterial type IV pilus assembly systems are involved in diverse functions including motility, adhesion and DNA uptake. Studies of the cyanobacterium *Synechococcus elongatus* sp. PCC7942 revealed that this machinery is also involved in suppression of biofilm formation: inactivation of components of this complex results in robust biofilm development. EbsA, a unique component of cyanobacterial type IV pilus assembly complexes, immunoprecipitates with a homolog of methionine γ-lyase (MGL). Here we demonstrate that MGL is required for biofilm development. Based on immunoprecipitation experiments using MGL as a bait, we suggest that this enzyme associates with a large enzymatic hub and with the translation machinery. Inactivation of *mgl* in the biofilm-forming mutant *pilB*Ω abrogates biofilm formation. However, assessment of expression of the *ebfG*-operon, which encodes proteins that comprise the biofilm matrix, using yellow fluorescent protein reporter strains, indicated high induction in the double mutant *pilB*/*mgl* similar to the biofilm-forming *pilB*-mutant. Thus, induction of the *ebfG*-operon is insufficient to promote biofilm development when a downstream step in which MGL participates is blocked. Overall, data suggest that MGL activity or an enzymatic activity of its interactor(s) is required to promote biofilm formation.

**Graphical Abstract:**

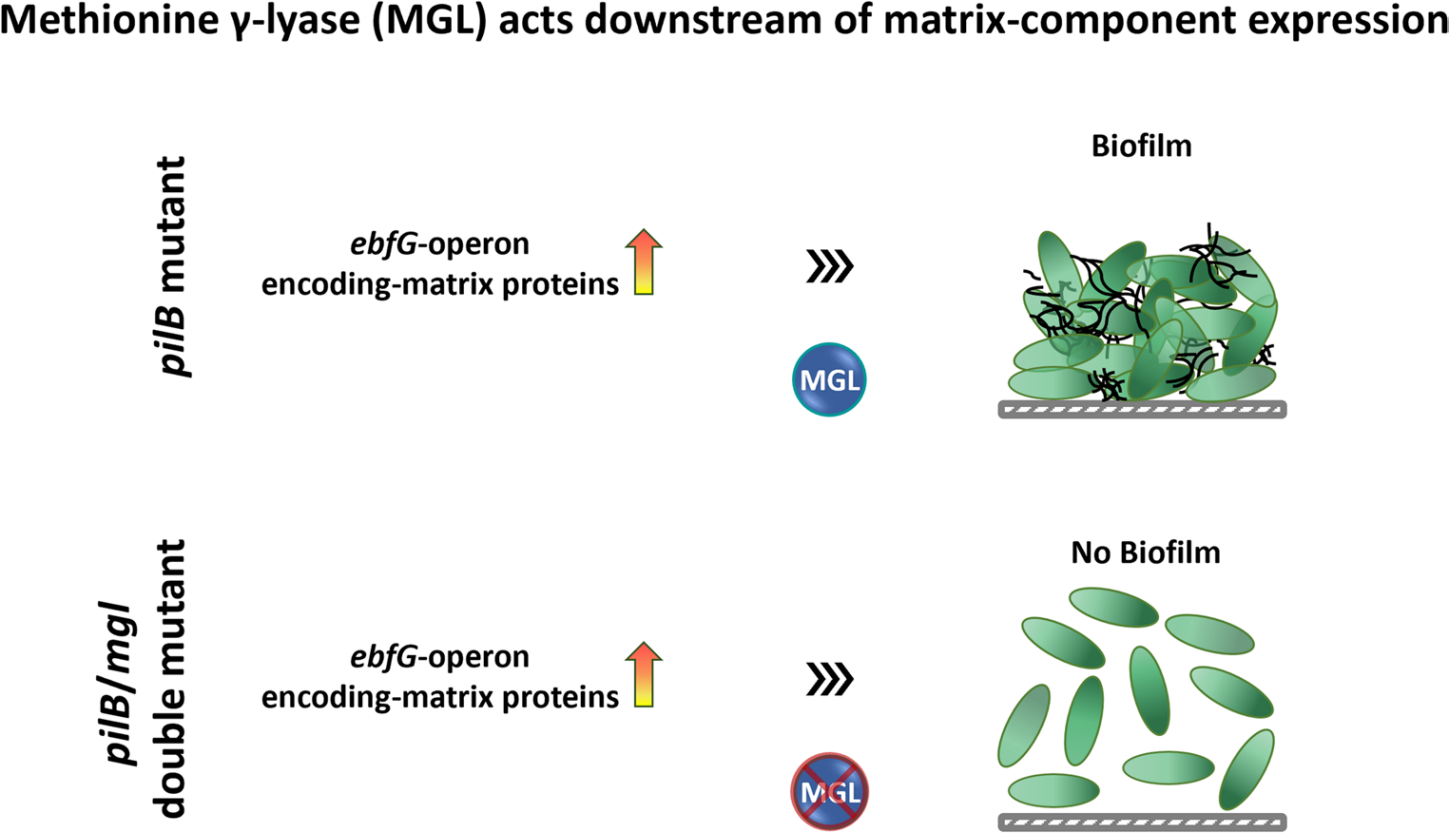

**Supplementary Information:**

The online version contains supplementary material available at 10.1007/s11274-025-04712-0.

## Introduction

Bacterial Type IV Pilus (T4P) assembly systems are specialized complexes that serve multiple functions including motility, adhesion, DNA uptake and interaction with host cells. Commonly, these machineries comprise 12–16 subunits located in the inner- and outer membranes as well as cytoplasmic and periplasmic components. Cytoplasmic-located ATPases drive rapid assembly and disassembly of the pili in response to environmental cues (Burrows 2012; Conradi et al. 2020; Costa et al. 2015; Ellison et al. 2022; McCallum et al. 2019; Schuergers and Wilde 2015; Wadhwa and Berg 2022). Cyanobacterial T4P systems include unique subunits that have not been reported to be part of T4P complexes in heterotrophic bacteria. One of these, a homolog of the RNA chaperone Hfq, interacts with the assembly ATPase, PilB (Schuergers et al. 2014; Yegorov et al. 2021). An additional component, dubbed EbsA (essential for biofilm suppression), is conserved in cyanobacteria, but homologs are not found outside this clade. Aside from its role in biofilm suppression in *Synechococcus elongatus* sp. PCC 7942 (hereafter *S. elongatus*), EbsA, which forms a tripartite complex with PilB and Hfq, is required for pilus assembly and DNA competence (Yegorov et al. 2021). Details of the mechanism in which EbsA takes part are, as yet, unknown.

Inactivation of *hfq* in *Synechocystis* sp. PCC 6803 (Dienst et al. 2008; Schuergers et al. 2014) or slr0038, encoding the EbsA homolog of this cyanobacterium (Yegorov et al. 2021), abolishes motility. Moreover, EbsA and Hfq are required for both motility and pilus extension in the filamentous cyanobacterium *Nostoc punctiforme* (Hassan et al. 2024). Thus, previous studies establish similar roles of EbsA and Hfq in diverse cyanobacteria.

**Fig. 1:**
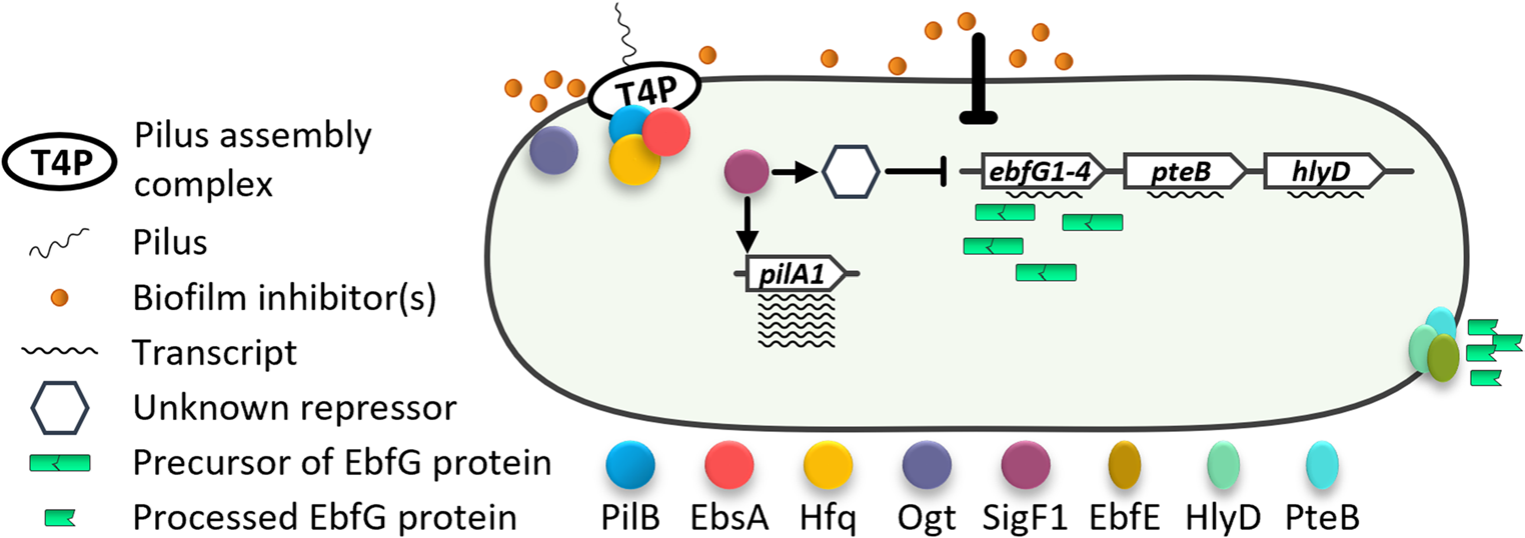
Expression of biofilm-enabling genes in ***S. elongatus*** is inhibited by a self- suppression mechanism. *Biofilm enabling components*: *ebfG*1–4, four small genes comprising the *ebfG*-operon, which encode matrix components. PteB, HlyD and EbfE are involved in secretion of EbfG proteins. *Biofilm inhibitory processes*: The type IV pilus (T4P) assembly complex is required for deposition of a biofilm inhibitor to the extracellular milieu, thereby suppressing transcription of the *ebfG*-operon, *pteB* and *hlyD*. PilB, the pilus assembly ATPase, forms a complex with EbsA and the RNA chaperon homolog Hfq. Ogt – a glycosyltransferase that glycosylates PilA1, the major pilus subunit. The RNA polymerase sigma factor SigF1 is involved in repression of biofilm-enabling genes by an extracellular pathway (thick T-bar) by regulating *pilA1*, as well as by an intracellular pathway (thin T-bar)

Cyanobacteria, photosynthetic prokaryotes that are known to form biofilms, contribute significantly to global photosynthesis (Falkowski 1994). The T4P complex of the model cyanobacterium *S. elongatus* plays a central role in biofilm regulation by depositing a biofilm inhibitor to the extracellular milieu (Fig. 1) (Nagar and Schwarz 2015; Nagar et al. 2017; Schatz et al. 2013; Simkovsky et al. 2022). Impairment of various T4P components including PilB, EbsA, and Hfq, as well as the glycosyltransferases Ogt, which glycosylates the major pilus subunit PilA (Suban et al. 2022), blocks the biofilm-suppression process, resulting in biofilm development. Recently, a sigma factor of RNA polymerase, SigF1, was demonstrated to play a pivotal role in the biofilm suppression process (Suban et al. 2024).

A few components that enable biofilm formation have also been identified – four small proteins denoted EbfG (*e*nable *b*iofilm *f*ormation with a *G*G-secretion motif) and their cognate type-I like secretion system comprising PteB, HlyD and EbfE (Parnasa et al. 2016, 2019). EbfG4 is a cell-surface and matrix protein as revealed by immunolocalization; EbfG1-3 are prone to amyloid formation and may thus be part of the biofilm matrix as well (Frenkel et al. 2023). The biofilm inhibitor – unidentified, but shown to be a molecule(s) of less than 1 kDa and resistant to proteases and autoclaving – suppresses transcription of the *ebfG*-operon, *pteB* and *hlyD*, thereby dictating planktonic growth under laboratory conditions (Parnasa et al. 2016; Simkovsky et al. 2022; Suban et al. 2024).

EbsA immunoprecipitates with numerous proteins (Yegorov et al. 2021) whose roles in biofilm development have not been investigated previously. This study employs a genetic approach to examine involvement of EbsA interactors in biofilm formation by *S. elongatus*. We demonstrate that gene synpcc7942_2562, which encodes a homolog of methionine γ-lyase (MGL) that interacts with EbsA, is required for biofilm formation. Moreover, inactivation of synpcc7942_2562 blocks the cascade of events that leads to biofilm formation downstream from induction of the *ebfG*-operon.

## Materials and methods

### Strains, culture conditions and biofilm assay

*S. elongatus*, an obligatory photoautotroph, and all derived strains were grown in mineral medium BG-11 as described (Sendersky et al. 2017). Cultures were grown at 30 °C in Pyrex tubes under bubbling with air enriched with 3% CO_2_ and illuminated by LED strip lights, 4000 K at flux of ~ 40 µmol photons m^− 2^ s^− 1^. Preparation of mutants and sequences of primers used in this study are provided in Supplementary Table 1. Mutants were constructed using individual plasmids from the uni-gene set (UGS) (Chen et al., 2012) and plasmid pAM5227 (pRP37) from S. Golden laboratory. All cloning products were validated by PCR analyses and sequencing. PCR was used to confirm complete chromosome segregation in all newly constructed strains.

Biofilm quantification in bubbled cultures is based on chlorophyll measurement as a proxy for biomass accumulation in sessile as well as in planktonic cells and representation of the relative fraction of chlorophyll in planktonic cells as previously published (Sendersky et al. 2017). To analyze biofilm-forming strains, an aliquot was collected from the upper part of the planktonic fraction, after which all remaining planktonic cells were carefully removed by aspiration using a 10 ml pipette. Detailed protocol for analysis of the percentage of chlorophyll in planktonic cells and chlorophyll extraction in 80% acetone and quantification based on absorbance at 663 nm was described previously (Sendersky et al. 2017).

### Flow cytometry and Immunoprecipitation

Flow cytometry was performed essentially as described (Frenkel et al. 2023; Suban et al. 2024). Analysis was performed following 6 days of growth, when robust biofilms were observed by *pilB*Ω. Previous analyses indicated that reporter expression did not change significantly between biofilm and planktonic cells (Frenkel et al. 2023; Suban et al. 2024); thus, in this study only the biofilm fraction of the *pilB*-mutant was measured. The planktonic cells were removed as described above for biofilm quantification, biofilms were resuspended in 1.5 ml BG-11 by rigorous pipetting, and 0.13 ml samples were transferred to 1.5 ml Eppendorf tubes for homogenization with a pellet pestle (Sigma-Aldrich, Z359971-1EA). The homogenized samples were filtered through a mesh (pore size 52 μm), supplemented with formaldehyde to a final concentration of 1%, diluted with phosphate-buffered saline to OD_750_ of ~ 0.0001, and measured using Becton Dickinson (BD) Laser Scanning Reflectance (LSR) Fortessa (excitation 488 nm, emission 530 ± 30 nm).

Analysis of flow cytometry data was performed, essentially as described (Yegorov et al. 2021). All statistical analyses were conducted in the statistical program R, version 3.3.2 (Team 2021). FCS files obtained from *FlowJo* were analysed with the *flowcore* package (Ellis, 2020). Mean, median and coefficient of variation (CV) of intensity distribution for each sample were calculated. Intensity values were log-transformed. Intensity distribution parameters for the different strains (mean, median and CV) were tested with 1-way ANOVA, followed by Tukey’s post hoc analysis.

Immunoprecipitation was performed essentially as described previously (Yegorov et al. 2021). A culture (250 ml; OD750 of 2.5 to 3) was concentrated by centrifugation to 2.2 ml, and a freshly prepared protease inhibitor cocktail (catalog number P8465-5ML; Sigma) was added to 3.44 mg/ml. Aliquots (1 ml) of the concentrated cell culture were combined with ~ 1 g of glass beads (catalog number 11079101; Biospec) in 2-ml Eppendorf tubes. Cells were broken using a mixer mill (catalog number MM400; Retch) at a frequency of 30 s21 for 2 min in prechilled holders (5 times, with 1 min of incubation on ice between the cycles). Cell lysates were centrifuged (relative centrifugal force [RCF] of 835_g for 5 min at 4 °C) to pellet the glass beads. The cell lysate (1 ml) was transferred to a 1.5-ml Eppendorf tube containing 100 µl washed anti-FLAG magnetic beads (catalog number M8823; Sigma) and incubated for 2 h at room temperature and overnight at 4 °C with mixing (RotoFlex; Argos Technologies). Beads were washed 4 to 5 times according to the manufacturer’s instructions, and elution was performed with 500 µl of 100 µM triple-FLAG peptide (catalog number A6001; APExBIO). Samples (10 µl) of eluates from each of the three independent biological repetitions were analyzed by SDS-PAGE and Western blot using anti-FLAG (Abcam, ab1162) and goat anti-rabbit (170–6515, Bio-Rad). SuperSignal™ West Pico PLUS Chemiluminescent kit (34580, Thermo Scientific). and the image was acquired using IBright FL1000 imaging system (Fig. S1).

Mass spectrometry analysis at the de Botton Institute for Protein Profiling at The Nancy and Stephen Grand Israel National Center for Personalized Medicine (Weizmann Institute of Science) was performed as previously described (Parnasa et al. 2016), except that trypsin digestion was not followed by chymotrypsin digestion and “discovery mode” was used rather than “targeted analysis.”

## Results

### Gene synpcc7942_2562 enables biofilm formation

**Fig. 2:**
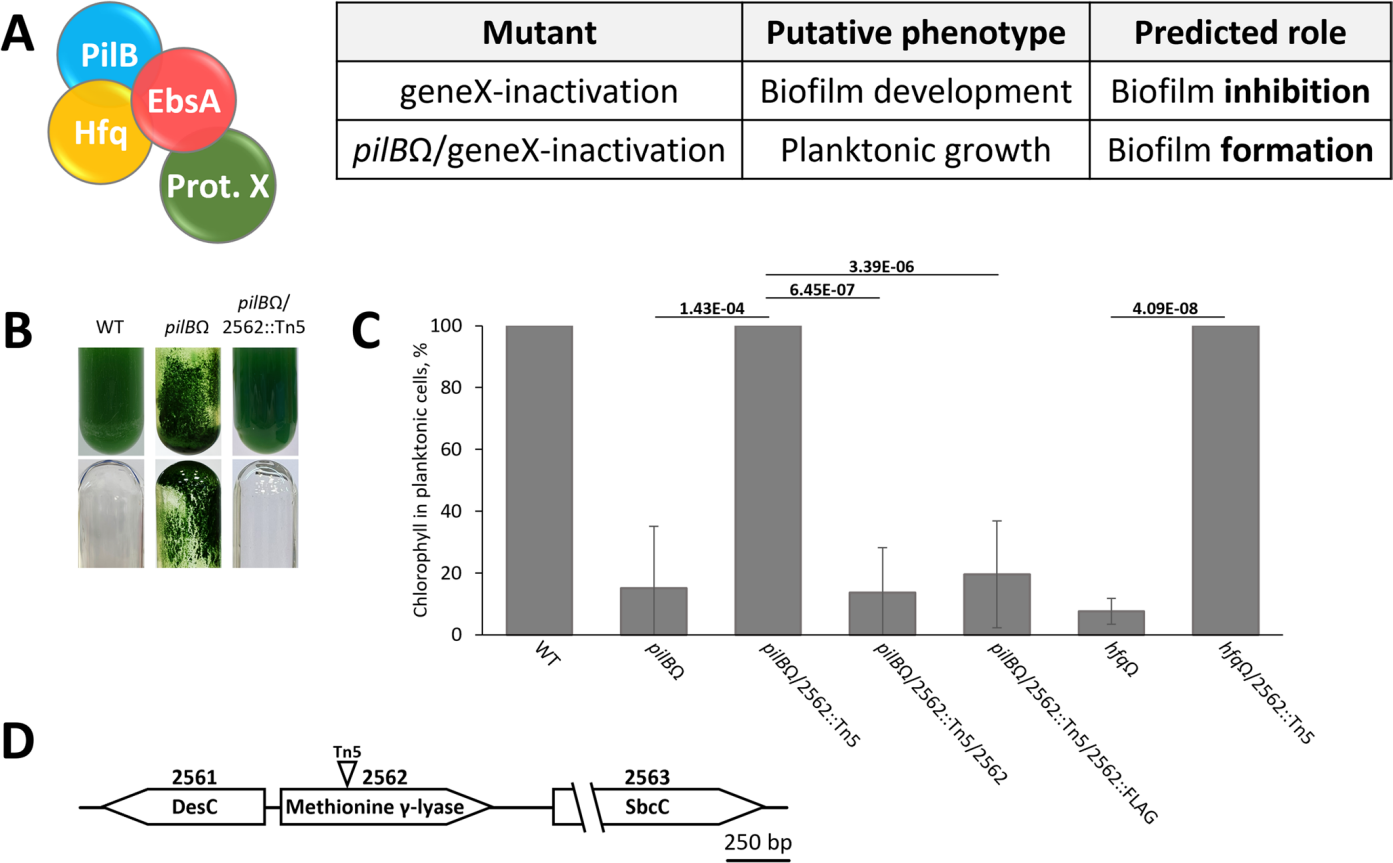
A genetic approach revealed requirement of gene synpcc7942_2562 for biofilm formation. **A**. Schematic representation of a particular EbsA-interactor (Prot. X) and outline of the genetic approach aimed at examining the role of EbsA interactors. EbsA is shown as part of the tripartite complex with PilB and Hfq. Single mutant – inactivation of the gene encoding Prot. X in wild type; Double mutant – gene inactivation in WT as well as *pilB* inactivation. **B**. Cultures of WT, *pilB*Ω and *pilB*Ω/2562::Tn5 before (top row) and after (bottom row) removal of the suspended cells. **C**. Percentage of chlorophyll in planktonic cells from total culture chlorophyll served to quantify biofilms. Strains analyzed: WT– wild type; *pilB*Ω– *pilB* inactivation using a spectinomycin resistance omega cassette; double mutant *pilB*Ω/2562::Tn5– gene synpcc7942_2562 was inactivated by a transposon cassette encoding kanamycin resistance along with *pilB* inactivation; *pilB*Ω/2562::Tn5/2562 and *pilB*Ω/2562::Tn5/2562-FLAG– double mutants that possess native synpcc7942_2562 gene and one encoding a FLAG-tagged protein, respectively; *hfq*Ω– *hfq* inactivation using a spectinomycin resistance omega cassette;*hfqB*Ω/2562::Tn5– a double mutant in which *pilB* and gene synpcc7942_2562 were inactivated. Significant changes (p-values; Student’s t-test, 2-tailed, type 3) are indicated for particular comparisons. Data show averages and standard deviation from three independent biological repetitions. **D**. Genomic region of synpcc7942_2562. Triangle indicates insertion point of the transposon cassette Tn5. Gene synpcc7942_2561 encodes delta-9 acyl-phospholipid desaturase (DesC); gene synpcc7942_2563 encodes an exonuclease (SbcC)

Our previous study identified 38 proteins that co-immunoprecipitate with the T4P component EbsA (fold > 6; p value < 0.05), including the protein encoded by synpcc7942_2562 that was enriched 11-fold (Yegorov et al. 2021). EbsA is essential for biofilm suppression, and interacts with proteins involved in biofilm inhibition (PilB and Hfq, Fig. 1); therefore, we tested the hypothesis that additional EbsA interactors take part in biofilm inhibition (Fig. 2A). Biofilm formation by a mutant defective for an interactor, in an otherwise WT background, should indicate its involvement in biofilm suppression. We prepared 27 single mutants, each inactivated in a gene encoding a particular EbsA interactor. These single mutants were grown individually and tested for biofilm formation; however, all mutants grew planktonically, as does the WT lab strain (Supplementary file 1). Therefore, we concluded that none of the examined EbsA interactors is essential for the biofilm suppression mechanism under the conditions tested.

We then tested the hypothesis that EbsA interacts with, and thus sequesters and prevents the activity of, a component that is *required* for biofilm formation. Because biofilm formation is constitutively repressed in the WT lab strain, a sensitized strain that relieves suppression was used to test this hypothesis. Inactivation of *pilB* is known to cause biofilm formation, so double mutants were constructed in which *pilB* was also inactivated (Fig. 2A). Planktonic growth of a double mutant (suppression of the *pilB* phenotype) would indicate involvement of the EbsA interactor in biofilm formation.

Of the 20 double mutants constructed and examined for biofilm development, only the strain in which *pilB* and gene synpcc7942_2562 were inactivated grew planktonically (Supplementary file 1, Fig. 2B). In this double mutant, *pilB*Ω/2562::Tn5, 100% of the chlorophyll is in planktonic cells similar to WT, in contrast to *pilB*Ω, which forms robust biofilms (Fig. 2C). Moreover, strain *pilB*Ω/2562::Tn5/2562, which carries an ectopic copy of gene synpcc7942_2562 in a neutral site in the genome, formed robust biofilms like *pilB*Ω (Fig. 2C). Restoration of biofilm formation by introduction of native synpcc7942_2562 to the double mutant validates the requirement of this gene for biofilm formation and negates any significant effect of the inserted Tn-cassette on neighboring genes. Additionally, introduction of a gene encoding a triple-FLAG-tagged version of the protein (2562::FLAG) to *pilB*Ω/2562::Tn5 also restored biofilm formation (Fig. 2C, strain *pilBΩ*/2562::Tn5/2562::FLAG), indicating that the tagged protein retains its function. Western blot analyses confirmed the presence of the FLAG-tagged protein (Fig. S1).

Additionally, we tested the effect of inactivation of gene synpcc7942_2562 in the biofilm-forming mutant, *hfq*Ω. The double mutant *hfq*Ω/2562::Tn5 grew planktonically (Fig. 2C). Thus, involvement of synpcc7942_2562 in biofilm formation is not unique to *pilBΩ*; rather, the gene product takes part in a biofilm-forming process common to *pilB*Ω and *hfq*Ω.

Measurements of total culture chlorophyll, an indicator for biomass accumulation, did not indicate significant changes between WT, *pilB*Ω, and *pilB*Ω/2562::Tn5 (Fig. S2).

A search for motifs using the InterPro server revealed that gene synpcc7942_2562 encodes a 409 AA protein that possesses an MGL domain, which encompasses most of the coding region (Fig. 2D). MGLs degrade sulfur-containing amino acids to α-keto acids, ammonia and thiols (Raboni et al. 2024; Sato and Nozaki 2009). For example, L-methionine is decomposed to methanethiol, ammonia and 2-oxobutanoate.

The UniProt database designates the protein encoded by synpcc7942_2562 “Aluminum resistance protein-like”; however, we could not find published articles supporting such a suggested role. Possibly, the name is derived from analysis based on screening of a barcoded transposon library included in the “Fitness Browser” website (Price et al. 2018), which indicates low fitness of synpcc7942_2562 mutants in the presence of aluminum chloride.

### Interactors of MGL are enriched in proteins involved in metabolic processes and translation components

Immunoprecipitation followed by mass spectrometry analysis was performed to identify proteins that associate with MGL and further investigate its role. Proteins co-precipitating with MGL may be its direct interactors or proteins that indirectly associate with MGL, as part of a large complex. The analysis revealed 61 proteins (fold ≥ 2; p value ≤ 0.05) that were significantly enriched in pull-down experiments performed with the 2562::Tn5/2562::FLAG strain, relative to the non-tagged strain (2562::Tn5/2562), which served as a negative control (Supplementary file 2). STRING analysis for these 61 proteins was used to test enrichment of particular functional groups. This analysis indicated enrichment of proteins involved in a variety of metabolic processes (Supplementary file 2) including 1,4-dihydroxy-2-naphthoyl-CoA synthase (MenB), involved in menaquinone synthesis, and ribose-5-phosphate isomerase A, participating in the pentose phosphate pathway. Components related to the translation machinery, including several tRNA ligases (e.g. ValS, MetG, AspS and GlyS), 23 S rRNA (Uracil-5-)- methyltransferase, elongation factor Ts, and translation initiation factors (IF-1 and IF-2) are also enriched in the pull-down. Based on these data we suggest that MGL is part of an enzymatic hub, which may be associated with the translation machinery.

Of note, EbsA was not detected among the proteins pulled down with MGL as bait. Each of the pull-down assays, using EbsA or MGL as bait, indicated numerous proteins, representing a large interactome and not necessarily direct interactions. We suggest that the stability of the interactome is affected by the particular bait and thus, do not expect to find complete consensus between the two pull-down data sets.

### MGL acts downstream of induction of the ebfG-operon

**Fig. 3:**
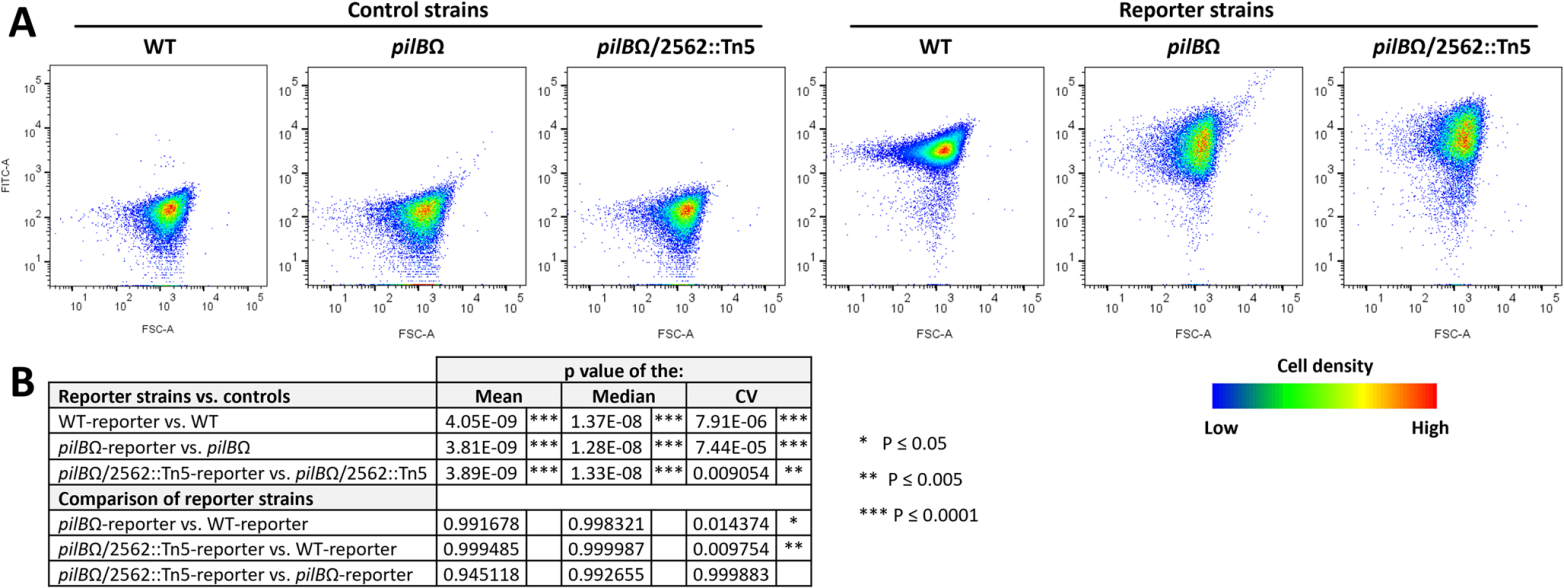
Flow cytometry analyses for control and reporter strains. **A**. Density plots showing YFP fluorescence (FITC-A) as a function of forward scattering (FSC-A). **B**. Summary of statistical analyses – shown are p values of the mean, median and the coefficient variation (CV)

To examine whether inactivation of synpcc7942_2562 affects transcription of the *ebfG*-operon, the products of which enable biofilm formation, we used a reporter construct in which the *ebfG* promoter drives expression of YFP (Frenkel et al. 2023). Briefly, a DNA fragment bearing the putative *ebfG*-operon promoter along with the 5’ untranslated region was attached to a yellow fluorescent protein (*yfp*) gene and cloned into neutral site I (Frenkel et al. 2023). This construct was used to create WT- *pilB*Ω- and *pilB*Ω/2562::Tn5-reporter strains. Analysis by flow cytometry was performed to assess YFP expression in these reporter strains and their cognate control strains (Fig. 3A). Gating was based on red autofluorescence (Fig. S3).

Fluorescence from all reporter strains differs significantly from their cognate negative control strains (Fig. 3B). Mean and median of YFP fluorescence did not significantly differ between *pilBΩ*- and WT-reporter strains. Fluorescence distribution, however, was wider in the *pilB*Ω-reporter, compared to WT-reporter (Fig. 3A), as manifested in significant difference in the coefficient variation (Fig. 3B, CV). Such data, which were reported previously, suggest that high expression of the *ebfG*-operon occurs only in a subfraction of the culture, which is sufficient to support biofilm formation by most cells in the culture (Frenkel et al. 2023; Suban et al. 2024).

Likewise, mean and median fluorescence of *pilB*Ω/2562::Tn5-reporter are similar to those measured for the WT-reporter; however, CV differs between these reporter strains (Fig. 3B). Furthermore, the mean and median fluorescence of the *pilB*Ω-reporter and the *pilB*Ω/2562::Tn5-reporter are not significantly different (Fig. 3B). Collectively, these data, which support high expression of the *ebfG*-operon in *pilB*Ω/2562::Tn5, suggest that MGL acts downstream from induction of this operon in the cascade of events that leads to biofilm formation.

## Discussion

Involvement of MGL in biofilm formation has been largely overlooked; however, one study reported that deletion of *mgl* in *Porphyromonas gingivalis* affected the multispecies composition of biofilms in vitro (Stephen et al. 2016). Our study indicates that the MGL homolog of *S. elongatus* is required for biofilm formation. The *ebfG*-operon, which encodes matrix components, is induced similarly in *pilB*Ω and *pilB*Ω/2562::Tn5, yet biofilm formation is completely abrogated in the double mutant. Thus, induction of the *ebfG*-operon is insufficient to promote biofilm development when a downstream step in which MGL participates is blocked. Thiols are produced upon MGL activity (Raboni et al. 2024; Sato and Nozaki 2009); thus, we hypothesize that inactivation of *S. elongatus mgl* may change the cellular redox status, thereby abrogating biofilm development.

The identities of additional proteins that co-precipitate with *S. elongatus* MGL suggest that this protein is part of an enzymatic hub that involves a variety of metabolic activities. Possibly, MGL impairment affects activity of additional enzymes associated with this interactome, consequently prohibiting biofilm development. Overall, the data indicate that MGL activity or enzymatic activity of its interactor(s) is required to promote biofilm formation. Additionally, co-immunoprecipitation data indicate enrichment of components related to the translation machinery; thus, absence of MGL may impair synthesis of particular proteins required for biofilm development.

## Supplementary Information

Below is the link to the electronic supplementary material.

Supplementary Material 1 (PDF 328 KB)

Supplementary Material 2 (XLSX 24.1 KB)

Supplementary Material 3 (XLSX 395 KB)

## Data Availability

No datasets were generated or analysed during the current study.
