# Supplementary material for: A homolog of methionine γ-lyase is required for biofilm development in the cyanobacterium Synechococcus elongatus
Source: World J Microbiol Biotechnol. 2025 Nov 25;41(12):475. doi: 10.1007/s11274-025-04712-0 (PMC12647202; doi:10.1007/s11274-025-04712-0)
Supplement: Supplementary file 1 — Supplementary Material 1 (PDF 328 KB) [file 11274_2025_4712_MOESM1_ESM.pdf]

Supplementary information

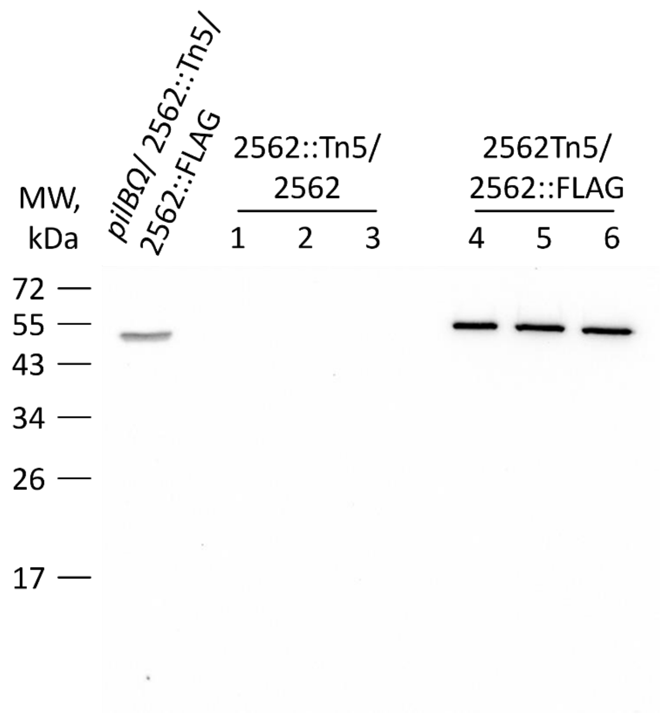

Figure S1: Eluates from immunoprecipitation experiments analyzed by Western blot. Immunoprecipitation from three independent biological repeats is shown for strains 2562::Tn/2562 (samples 1-3) and 2562::Tn/2562::FLAG (samples 4-6). Each lane represents 2% of the eluate. Cellular extract representing 2 μg chlorophyll from strain *pilB*Ω/2562::Tn/2562::FLAG is also included. The image was acquired using IBright FL1000 imaging system.

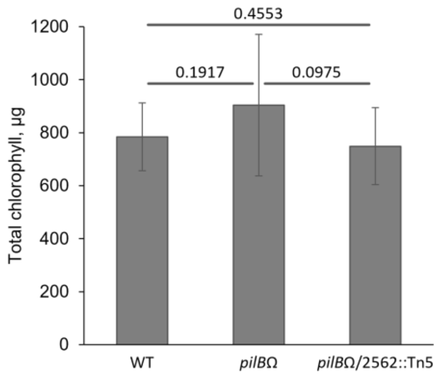

Figure S2: Total chlorophyll in cultures of WT, *pilB*Ω and *pilB*Ω/2562::Tn5. Data represent averages and standard deviations from six independent biological repeats. p-values; Student's t-test (2-talis, type 3) are indicated.

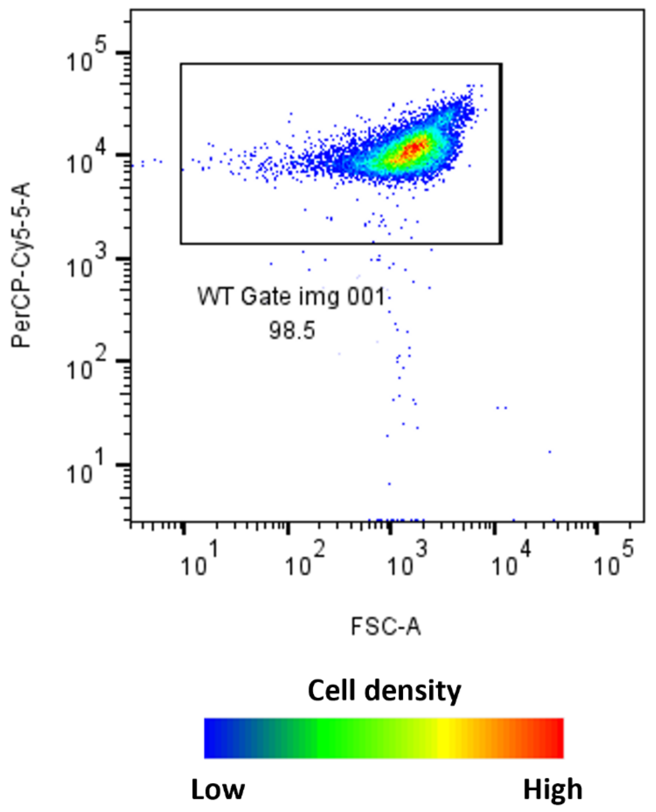

Figure S3: Gating for flow cytometry analysis based on cyanobacterial red autofluorescence. Density plots showing PerCP-Cy5-5-A fluorescence (excitation 488nm, emission 645 ± 25 nm) as a function of forward scattering (FSC-A).

Table S1: Summary of mutant generation and primers used in this study.

Gene disruption and strain construction	Description
2562::Tn5, insertional inactivation of synpcc7942_2562	UGS vector 17D9 used for inactivation, bears Tn5 transposon inserted 334 bp downstream of the start codon of 2562. Inserted fragment confers kanamycin resistance.
*pilB*Ω/2562::Tn5	Strain 2562::Tn5 was transformed with a plasmid leading to *pilB* inactivation by insertion of an Ω cassette conferring spectinomycin resistance [1].
*hfq*Ω/2562::Tn5	Strain 2562::Tn5 was transformed with a plasmid leading to *hfq* inactivation by insertion of chloramphenicol resistance cassette that is based on the Ω inactivation fragment [2].
**Primers**	
**Purpose**	**Sequence (upper forward, lower reverse)**
**Cloning of synpcc7942_2562 for complementation**	
Native synpcc7942_2562	AAGACCAGCCATTGCCAACC
	TTCAAGCGGGACCGATCG
FLAG-tagged synpcc7942_2562	AAGACCAGCCATTGCCAACC
	CTACTTATCGTCGTCATCCTTGTAATCGATATC GTGATCCTTGTAATCCCCATCGTGATCCTTGTA ATCAGCGGGACCGATCGCCGC
Examine segregation of insertions in neutral site 1	CGTCGAAGATGGAAAAGCTC
	ATTGACCCGGTAGGGATTTC
**Primers used to confirm segregation**	
synpcc7942_0221	ATGGAACCGCGAAGCTGACG
	AGGGCTGGATTTTCTTGGTCG
synpcc7942_0255	TTGGCAGCGCATCCATATCG
	TCAATGCCGTGCAGTTGTCC
synpcc7942_0437	ATCCTTGCACCACAGTAATGG
	ATCGAGATCAACTGCAGCTTGG
synpcc7942_ 0501	ACATTCAGTACGAGCGCTGG
	AATTTCGACGATCCGGTTGG
synpcc7942_0730	AATGCAGTCACAGCCTAGG
	TTACCCGCACGTCTTCAGC
synpcc7942_0772	AGAGCAGGCCAATGCCTTGC
	ACCTGTTTCGGGATCAATGCC
synpcc7942_0788	ACTTCAAGGGTCTGGAGTATCG
	AAGGTTGAGACGAGCAGTGC
synpcc7942_0863	AAGGTGTAGAAGCAACCG
	CCTGATCGATGAAGACGTTC
synpcc7942_0864	TTGAGCCAGTCCATCGACC
	AACGACAGGTTTCCAAGAGC
synpcc7942_0998	AACTGGTTGAGGGTGTTCGC
	TTGCAGTTGCACCCGAGC
synpcc7942_1071	ATGAAAGTGGCCGGCATTGC
	ATCGAAGCCTCAGCAATCACC
synpcc7942_1106	TTGCCGGAGTTGAAGACTGC
	AAGTCATAGGAGTGAGGGC
synpcc7942_1216	ACTTCCGCTGAGATGACTAGC
	ACGTTATCGGAGACAAATTCC
synpcc7942_1390	AGCTAGATTCACCACAACCC

	TTGGCAATTGGTTGCTTGGG
synpcc7942_1412	ATTACTGGTGCCAAAGGCTTGC
	AGCGAAACTTCCAGATCGTGC
synpcc7942_1775	TAATTGCTAGCTGGGGTGCC
	TTGGATGTAGCGTTGCTGGC
synpcc7942_1813	ATGGCGATTCTAGAGCAAGG
	ATGATAGGCTGATCAGCTCC
synpcc7942_1919	ATTGATAGTCAGCAGCGAGC
	ACTCATCTCCCTGCACATGC
synpcc7942_1965	ATGAGCACCGAGATTGCGC
	ACCGACTTCATGGATGACCC
synpcc7942_2008	ACCTTGCTGCGCAAAATTCC
	AGGGCCTCTTCCAAAATTGG
synpcc7942_2063	TTACCAGCCGTGCGATCG
	TTGACGCAGTCCGAAGGC
synpcc7942_2068	AACCTAGTGCTACGCCTGC
	TTCCCAGTGATCGTCGTTGG
synpcc7942_2073	TTACGGACTCGATAAGCTGC
	ATCGTGGTTGTCGGCAGGG
synpcc7942_2297	AACCCTTCTCTGATCACAGC
	ACCAATCGAGGATGCGACC
synpcc7942_2380	AACGCCCAAATCCTGTCAGG
	ACCATTGACAGAGCCTTGCG
synpcc7942_2521	ATCCAATGGCACTGTTTCGC
	AGAGCCCTGTTGCATCAGC
synpcc7942_2562	ATGGCCACGATGATCTCGG
	ATCAATACTGAGGCTGCGTCG

References for Table 1:

1. Schatz, D., E. Nagar, E. Sendersky, R. Parnasa, S. Zilberman, S. Carmeli, Y. Mastai, E. Shimoni, E. Klein, O. Yeger, Z. Reich, and R. Schwarz, *Self-suppression of biofilm formation in the cyanobacterium Synechococcus elongatus.* Environ Microbiol, 2013. **15**(6): p. 1786-94.
2. Yegorov, Y., E. Sendersky, S. Zilberman, E. Nagar, H. Waldman Ben-Asher, E. Shimoni, R. Simkovsky, S.S. Golden, A. LiWang, and R. Schwarz, *A Cyanobacterial Component Required for Pilus Biogenesis Affects the Exoproteome.* mBio, 2021. **12**(2).
